# Analysis of the effect of adjuvant radiotherapy on outcomes and complications after radical hysterectomy in FIGO stage IB1 cervical cancer patients with intermediate risk factors (GOTIC Study)

**DOI:** 10.1186/s12957-016-0931-4

**Published:** 2016-06-29

**Authors:** Kazuto Nakamura, Yoshikazu Kitahara, Toyomi Satoh, Yuji Takei, Masashi Takano, Shoji Nagao, Isao Sekiguchi, Mitsuaki Suzuki

**Affiliations:** Department of Obstetrics and Gynecology, Gunma University Hospital, Maebashi, Gunma 371-8511 Japan; Faculty of Medicine, University of Tsukuba, Tsukuba, Ibaraki 305-8577 Japan; Department of Obstetrics and Gynecology, Jichi Medical University Hospital, Shimotsuke, Tochigi 329-0498 Japan; Department of Obstetrics and Gynecology, National Defense Medical College, Tokorozawa, Saitama 359-8513 Japan; Department of Gynecologic Oncology, Saitama Medical University International Medical Center, Hidaka, Saitama 350-1298 Japan; Department of Gynecology, Tochigi Cancer Center, Utusnomiya, Tochigi, 320-0834 Japan; Current address: Department of Gynecology, Gunma Cancer Center, Ota, Gunma 373-8550 Japan; Current address: Department of Gynecology, Hyogo Cancer Center, Akashi, Hyogo 673-8558 Japan

**Keywords:** Cervical cancer, Radiation, Adverse event

## Abstract

**Background:**

There are no definitive criteria for identifying which patients with The International Federation of Gynecology and Obstetrics (FIGO) stage IB cervical cancer will benefit from adjuvant therapy after radical hysterectomy. The aims of this study were to clarify the efficacy of adjuvant therapy and assess complications after radical hysterectomy in patients with FIGO stage IB1 cervical cancer with intermediate risk factors.

**Methods:**

Between January 2005 and December 2009, the medical records of 75 stage IB1 patients’ intermediate risk factors (i.e., tumor size 2–4 cm, lymphovascular involvement, and/or deep stromal invasion >1/2) who underwent radical hysterectomy at six institutions were collected, and these patients were enrolled in this nonrandomized retrospective study. We simplified the criteria of intermediate risk factors as much as possible, as the criteria adopted in some clinical studies are complicated in practice.

**Results:**

The patients were grouped according to the receipt of adjuvant therapy as follows: 46 patients, no further treatment; 19 patients, external beam radiation treatment, including 9 patients who received brachytherapy; 5 patients, concurrent chemoradiotherapy (CCRT); and 5 patients, chemotherapy (CT). The clinical outcomes and complications in each group were analyzed. After an average follow-up of 82.6 months (range, 24–135 months), only one patient with all three risk factors who received radiotherapy (RT) experienced recurrence. Excluding this patient, the remaining patients who received RT, CCRT, or CT had two or three risk factors. Lymphedema was significantly more common among patients who received RT or CCRT, whereas the incidence of ileus and ureteral obstruction was not different among the treatment groups. However, an unsutured peritoneum increased the risk of ileus.

**Conclusions:**

The findings of this study suggest that RT and CCRT after radical hysterectomy are not beneficial in patients with intermediate risk factors. In particular, RT and CCRT appeared to increase the incidence of lymphedema. A prospective randomized study is needed to verify the findings of this study.

## Background

Cervical cancer is the fourth most common cancer among women worldwide and the second most diagnosed cancer in developing countries [1]. Radical hysterectomy with pelvic lymphadenectomy has been a primary treatment in women with stage IB cervical cancer, and the procedure is associated with a 5-year survival rate of 87–92 % [2]. Radiotherapy (RT) is a feasible technique that provides similar outcomes as radical hysterectomy [2–4]. Surgery enables pathological examination by surgeons, permitting identification of risk factors for cancer recurrence. In general, patients with parametrial invasion, a positive vaginal margin, or positive pelvic lymph nodes who are diagnosed as being at high risk are assigned to receive adjuvant therapies. By contrast, large tumor size, deep stromal invasion, and lymphovascular invasion are classified as intermediate risk factors, and adjuvant therapy for patients with these risk factors remains controversial. Although the criteria for intermediate risk factors defined by the Gynecologic Oncology Group (GOG) study [5] have been widely accepted, each institution offers adjuvant therapy to patients based on its own protocol. Thus, there is always a problem of interpreting discrepant results between studies because of the different study models. On the contrary, it has been recognized that postoperative RT results in a significant increase in the incidence of adverse events affecting quality of life, such as lymphedema, ileus, and ureteral obstruction [6–8].

The GOG study concluded that pelvic RT after radical hysterectomy significantly improves progression-free survival and benefits patients with histological types of adenocarcinoma and adenosquamous carcinoma [9]. The aims of the current retrospective study were to evaluate the effect of RT and treatment-related morbidity after radical hysterectomy for patients with intermediate risk as defined by simplified criteria.

## Methods

Between 2005 and 2009, the medical records of 89 stage IB1 patients with intermediate risk factors (i.e., tumor size 2–4 cm, lymphovascular involvement, and/or deep stromal invasion >1/2) who underwent type III radical hysterectomy as defined by Piver et al. [10] and bilateral pelvic lymphadenectomy were obtained for this retrospective study from six institutions belonging to the Gynecologic Oncology Trial and Investigation Consortium of North Kanto (GOTIC): Gunma University, Tsukuba University, Jichi Medical University, National Defense Medical College, Saitama Medical University International Medical Center, and Tochigi Prefectural Cancer Center. None of the patients had received preoperative treatment such as neoadjuvant chemotherapy (CT) or RT. Informed consent was not obtained from each participant because this was a retrospective study. Instead of that, all participants were given the right to withdraw the use of the data. The protocol of this study was approved based on the necessity of the individual institutions’ ethical committees.

Based on each institution’s criteria, patients received adjuvant therapy, including RT, CT, or concurrent chemoradiotherapy (CCRT). RT consisted of conventional external beam (EBRT) to the pelvis (28–42 Gy) in fractions of 1.8–2.0 Gy for 28–42 days. Nine patients in the RT group also received vaginal brachytherapy (BRA) in fractions of 4–7 Gy for a total dose of 7–21 Gy. In the CCRT group, cisplatin (40 mg/m^2^) was infused intravenously every week. In the CT group, patients received paclitaxel (175 mg/m^2^) plus carboplatin (AUC 6) every 3 weeks for 6 cycles.

Data regarding tumor size (MS), histopathological findings, depth of stromal invasion [11], and lymphovascular space invasion (LVSI) are summarized in Table 1. All patients received regular follow-up. During follow-up, complications such as ileus and ureteral obstruction were classified according to the Common Toxicity Criteria. The severity of lymphedema was rated according to the staging system of the International Society of Lymphology as follows: stage 0, a latent or sub-clinical condition in which swelling in not evident; stage 1, temporary visible swelling that can be reduced by elevation of the limb; stage 2, clear pitting and limb elevation cannot reduce tissue swelling; and stage 3, also known as lymphostatic elephantiasis, tissue becomes extremely swollen, leading to skin changes such as acanthosis, fat deposits, and warty overgrowths [12].

**Table 1:** Patient characteristics (*n* = 75)

	RT/CCRT					
	NFT	EBRT	EBRT + BRA	EBRT (CCRT)	EBRT + BRA (CCRT)	CT
	*n* = 46	*n* = 10	*n* = 9	*n* = 2	*n* = 3	*n* = 5
Age (years)	46.8 (27–78)	44.7 (27–67)	49.9 (31–69)	63.5 (55–72)	38.0 (33–43)	53.0 (34–66)
Follow-up time (months)	78.7 (51–118)	90.6 (64–117)	77.8 (24–120)	98.0 (61–135)	113.3 (110–117)	86.8 (61–134)
Tumor size						
<2 cm	6 (13.0 %)	0 (0.0 %)	0 (0.0 %)	0 (0.0 %)	0 (0.0 %)	0 (0.0 %)
2 cm ≤ tumor < 4 cm	40 (87.0 %)	10 (100.0 %)	9 (100.0 %)	2 (100.0 %)	3 (100.0 %)	5 (100.0 %)
Histologic type						
Squamous	31 (67.4 %)	10 (100.0 %)	9 (100.0 %)	1 (50.0 %)	0 (0.0 %)	0 (0.0 %)
Adenocarcinoma	12 (26.1 %)	0 (0.0 %)	0 (0.0 %)	1 (50.0 %)	2 (66.7 %)	5 (100.0 %)
Adenosquamous	3 (6.5 %)	0 (0.0 %)	0 (0.0 %)	0 (0.0 %)	1 (33.3 %)	0 (0.0 %)
Stromal invasion						
<1/2	24 (52.2 %)	3 (30.0 %)	2 (22.2 %)	0 (0.0 %)	0 (0.0 %)	0 (0.0 %)
≥1/2	22 (47.8 %)	7 (70.0 %)	7 (77.8 %)	2 (100.0 %)	3 (100.0 %)	5 (100.0 %)
Lymphovascular invasion						
–	37 (80.4 %)	1 (10.0 %)	3 (33.3 %)	0 (0.0 %)	1 (33.3 %)	1 (20.0 %)
+	9 (19.6 %)	9 (90.0 %)	6 (66.7 %)	2 (100.0 %)	2 (66.7 %)	4 (80.0 %)

*Abbreviations*: *NFT* no further treatment, *RT* radiation therapy, *CCRT* concurrent chemoradiotherapy, *EBRT* external beam radiation therapy, *BRA* brachytherapy, *CT* chemotherapy

Statistically significant differences in the severity of adverse events, namely lymphedema, ileus, and ureteral obstruction, as a result of no further treatment (NFT, *n* = 46), RT and CCRT (*n* = 24), or CT (*n* = 5) were analyzed using the Kruskal-Wallis or chi-squared test. All tests were two-tailed, and a *P* value <0.05 was considered statistically significant.

The presence or absence of complications was determined, and cross-tabulation was performed when the retroperitoneum was opened or closed during surgery and when RT was or was not administered. In addition, odds ratios and 95 % confidence intervals for the presence or absence of complications were calculated using logistic regression analyses based on whether the retroperitoneum was opened or whether RT was performed.

JMP ver. 9 (SAS Institution Japan Inc., Tokyo, Japan) was used for all analyses.

## Results

Eighty-nine patients were enrolled in this retrospective study from six institutions; meanwhile, 14 patients were excluded because of incompatibility with the inclusion criteria and incomplete follow-up data. Overall, the study included 51, 20, and 4 patients with squamous cell carcinoma, adenocarcinoma, and adenosquamous histology, respectively (Table 1). One patient with all three intermediate risk factors who received EBRT and BRA died of a recurrent tumor in the lungs 24 months later. All other patients are alive without recurrence, and they have been followed up for an average of 84.3 months (range, 47–135 months).

Table 2 shows the characteristics of intermediate risk factors in this group of patients. Among the 75 patients, 24 received either RT or CCRT and 5 were treated with CT alone. Only a single patient who had one intermediate risk factor received EBRT and BRA. By contrast, 19/24 (79.2 %) patients who had all three intermediate risk factors as defined in this study received adjuvant therapy.

**Table 2:** Pathological characteristics of intermediate risk cervical cancer

Risk factor	Adjuvant therapy		
	NFT	RT/CCRT	CT
	*n* = 46	*n* = 24	*n* = 5
MS	18	1	0
SI	4	0	0
LVSI	1	0	0
MS + SI	12	4	1
MS + LVSI	5	4	0
SI + LVSI	1	0	0
MS + SI + LVSI	5	15	4

*Abbreviations*: *NFT* no further treatment, *RT* radiation therapy, *CCRT* concurrent chemoradiotherapy, *CT* chemotherapy, *MS* mass size (2 ≤ tumor < 4 cm), *SI* stromal invasion, *LVSI* lymphovascular space involvement

Lymphedema was observed more frequently in patients who received postoperative RT than in those who did not receive RT or CT (*P* < 0.001). The risks of ileus and ureteral obstruction were not significantly different between patients who received NFT/CT or RT/CCRT. Other radiation-related adverse effects such as cystitis and proctitis were extremely rare (Table 3). We further assessed the severity of lymphedema associated with RT, finding that more patients who receive RT developed grade 2 or 3 lymphedema (*P* < 0.001) (Table 4).

**Table 3:** The frequency of complications by treatment regimens

	Treatment			*P* value^a^
	NFT (*n* = 46)	RT/CCRT (*n* = 24)	CT (*n* = 5)	
Lymphedema	8	14	0	0.001
Ileus	9	4	0	0.544
Ureter obstruction	1	2	1	0.184
Radiation cystitis		0		–
Radiation proctitis		2		–

Lymphedema level is rated according to the staging system by the International Society of Lymphology

*Abbreviations*: *NFT* no further treatment, *CT* chemotherapy, *RT* radiation therapy

^a^Chi-squared test

**Table 4:** Incidences of lymphedema grades by treatment regimens

	Treatment			*P* value^a^
	NFT (*n* = 46)	RT/CCRT (*n* = 24)	CT (*n* = 5)	
Lymphedema				0.001
Grade 0	38	10	5	
Grade 1	7	7	0	
Grade 2	1	6	0	
Grade 3	0	1	0	

Lymphedema level is rated according to the staging system by the International Society of Lymphology

*Abbreviations*: *NFT* no further treatment, *CT* chemotherapy, *RT* radiation therapy

^a^Kruskal-Wallis test

**Fig. 1:**
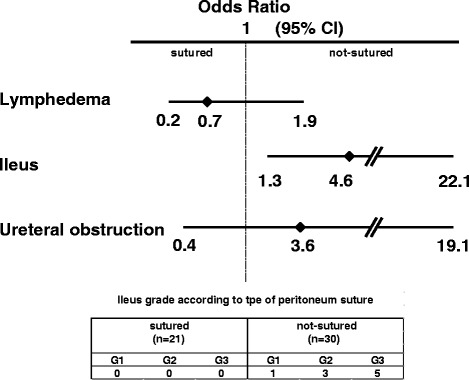
Adverse events for patients with an unsutured pelvic peritoneum who did not receive radiotherapy. *Horizontal lines* represent odds ratio (*diamonds*) with 95 % confidence intervals (CIs)

Retroperitoneal suturing after lymphadenectomy was also assessed to evaluate the prophylactic effect of an unsutured pelvic peritoneum on lymphedema risk. The overall lymphedema rate was comparable between patients with an unsutured pelvic peritoneum and those with a sutured pelvic peritoneum (Table 5). However, an unsutured pelvic peritoneum was significantly associated with ileus among patients who did not receive adjuvant RT (Fig. 1).

**Table 5:** Adverse events in unsutured and sutured peritoneum status

	Not sutured (*n* = 36)	Sutured (*n* = 39)
Radiation (−)		
Ileus (−)	21	22
Ileus (+)	9	0
Lymphedema (−)	24	19
Lymphedema (+)	6	3
Radiation (+)		
Ileus (−)	5	14
Ileus (+)	1	3
Lymphedema (−)	3	7
Lymphedema (+)	3	10

## Discussion

The outcome of surgery with or without adjuvant RT in patients with early-stage cervical cancer has been debated by many researchers. In general, RT has been demonstrated to significantly decrease the risk of local recurrence. However, overall survival is not conclusively improved, whereas certain subset analyses illustrated a positive effect of RT on the reduction of recurrence, for example, the GOG 92 study suggested that RT is effective for patients with a combination of deep stromal invasion and large tumor size (≥4 cm) [9]. Tumor size ≥4 cm has been strongly correlated with recurrence [13–17]. As we assumed that tumor size ≥4 cm was a high risk factor even in the intermediate risk group, we excluded patients with tumor diameters ≥4 cm in this study. It is also accepted that stromal invasion by tumors is an important prognostic factor, and a majority of studies evaluated invasion depth via measurements in absolute millimeters or fractions of muscle layer [9, 17]. Stromal invasion in fractional seconds was used in this study because variable thickness in the cervical wall might not reflect the extent of stromal invasion. In addition, measurement in fractional thirds appeared difficult, especially in patients with a thin cervical wall, and it might increase discrepancies because of the multi-institutional nature of the study. Previously, a combination of intermediate risk factors, such as large tumor size, LVSI, and stromal invasion, was associated with incremental recurrence rates of up to 15–20 % [5, 13, 16, 17]. In this study, only one patient (1/24) with a single risk factor received adjuvant RT, whereas 28/51 patients with multiple risk factors received adjuvant therapy (Table 2). However, this study has some limitations, including its retrospective nature and the possible diversity of treatment modalities among institutions, resulting in heterogeneity among the treatment groups. For example, the introduction of CCRT for treating high risk cervical cancer probably influenced some institutions to utilize CCRT, as 4/5 patients who received CCRT had all three risk factors (Table 1), which may result in bias in interpreting the results. In this study, only one patient with all three intermediate risk factors who received RT and BRA died of recurrent tumor in the lungs 24 months later. This finding suggests that adjuvant RT does not benefit patients with intermediate risk factors as defined in this study. These issues can be addressed via a randomized design study in the future.

Another purpose of this study was to assess adverse events associated with RT, as adjuvant RT has long been known to increase complications [18, 19]. Adjuvant CT alone for post-radical hysterectomy patients has been revealed to provide a better postoperative quality of life by eliminating RT-related morbidities such as small-bowel obstruction or leg edema [6, 8], as also supported by our results (Table 3). Based on the small number of cases of ileus and ureteral obstruction, there was no difference between NFT/CT and RT/CCRT, which was consistent with a meta-analysis [11] of two combined trials by Bilek et al. [20] and GOG 92 [9]. Conversely, the incidence of lymphedema was significantly higher and the adverse event was of greater severity for patients who received RT or CCRT (*P* < 0.001). Several studies indicated that an unsutured peritoneum after pelvic lymphadenectomy reduced the risk of lymphocyst formation [21, 22]. In this study, we assessed whether the incidence of lymphedema improved when the retroperitoneum was left open. In contrast to a previous report suggesting that an unsutured peritoneum significantly reduced the risk of lymphedema [23], this strategy did not provide a significant advantage in avoiding lymphedema in this study (Table 5). Lymphedema is most commonly diagnosed within the first year, but a certain number of patients manifest symptoms in later years [24, 25]. Thus, the possible reason for discrepant results between studies may be attributable to the follow-up duration; specifically, the average observation period was 82.6 months in our study, whereas other studies evaluated patients for 3 years after surgery. Intriguingly, our study demonstrated that an unsutured peritoneum without RT significantly increased the incidence of ileus, whereas there was no difference between an unsutured and sutured peritoneum regarding the risks of lymphedema and ureteral obstruction (Fig. 1).

## Conclusions

In the present study, postoperative adjuvant RT significantly increased adverse events for intermediate risk patients as defined in this study. To date, no trial has established a solid consensus regarding RT after surgery for early-stage cervical cancer. Although this retrospective study included a limited number of patients, the results provide useful information for further consideration in the management of patients with intermediate risk cervical cancer.

## Abbreviations

BRA, brachytherapy; EBRT, external beam radiation therapy; CCRT, concurrent chemoradiotherapy; CT, chemotherapy; LVSI, lymphovascular space invasion; MS, tumor size; NFT, no further treatment; RT, radiation treatment; SI, stromal invasion
